# Cardioprotective therapies for ST-elevation myocardial infarction: the emerging role of thyroid hormone: a narrative review

**DOI:** 10.3389/fendo.2025.1696749

**Published:** 2025-12-15

**Authors:** Konstantinos Grigoriou, Paschalis Karakasis, Vasileios Lamprou, George Michas, Konstantinos Pamporis, Athanasios Trikas, Constantinos Pantos, Iordanis Mourouzis

**Affiliations:** 1 Department of Pharmacology, University of Athens, Athens, Greece; 2 Second Department of Cardiology, Hippokration General Hospital, Aristotle University of Thessaloniki, Thessaloniki, Greece; 3 Manchester Heart Centre, Manchester Royal Infirmary, Manchester University National Health Service (NHS) Foundation Trust, Manchester, United Kingdom; 4 Department of Cardiology, Evangelismos General Hospital of Athens, Athens, Greece

**Keywords:** thyroid hormone, triiodothyronine, thyroxine, cardioprotective strategies, myocardial infarction, infarct size, post-ischemic cardiac remodeling

## Abstract

The mortality rates and the incidence of cardiac remodeling and subsequent heart failure remain high, despite ongoing advancements in the management of patients with ST-segment elevation myocardial infarction (STEMI). Most of the adjunctive therapies aiming to further reduce myocardial infarction (MI) size have failed to apply in daily clinical practice. In this context, new promising therapeutic approaches aiming to enhance myocardial salvage have emerged. Recent studies have suggested that thyroid hormone (TH) may have regenerative effects on ischemic myocardium. Immediate treatment with TH appears to trigger repair and the regeneration process in the injured myocardium, especially in patients with large infarct sizes. The aim of this narrative review is to summarize the most recent advances in the use of TH for salvaging ischemic myocardium following STEMI and place it among the most promising cardioprotective therapies. Emphasis is placed on preclinical and clinical data that highlight the favorable effects of TH in enhancing myocardial recovery and improving outcomes after acute myocardial ischemia.

## Introduction

Despite significant advances in cardiovascular diagnosis and treatment, cardiovascular disease (CVD) remains the leading cause of mortality and morbidity worldwide (1). Ischemic heart disease (IHD) is the most common cause of CVD related death, accounting for 44% of all CVD deaths in males and 38% in females (2). In the context of ST-segment elevation myocardial infarction (STEMI), coronary reperfusion strategies, such as coronary artery bypass grafting (CABG) and percutaneous coronary angioplasty (PCI), along with anticoagulant and antiplatelet agents, represent the main therapeutic options (3). In patients with STEMI, timely reperfusion of the occluded vessel is the most significant intervention to reduce infarct size and salvage viable myocardium. However, the incidence of heart failure (HF) and death remain high due to factors such as ischemia-reperfusion injury (IRI), cardiac remodeling, and microvascular obstruction (4–6).

Despite extensive efforts to translate various cardioprotective strategies into clinical practice, currently there is no established therapy to reduce infarct size following an acute coronary syndrome (ACS) (7, 8). However, novel cardioprotective therapies -including two that have received FDA approval- have emerged and are considered promising. Thyroid hormone (TH) administration appears to play a crucial role in the myocardium’s response to stress following acute myocardial injury. Immediate treatment with TH has been shown to limit the extent of ischemic damage and trigger the repair/regeneration process of the injured myocardium (9, 10).

In this narrative review, we provide a brief overview of the previously tested treatment strategies aiming to reduce infarct size in the setting of STEMI and we include an extensive analysis of preclinical and clinical data of the TH as both a risk factor and a potential agent that can prevent further myocardial injury.

## Methods

The present review mainly aims to investigate the role of TH in promoting myocardial recovery, reducing infarct size, and preventing cardiac remodeling following acute myocardial ischemia. A comprehensive literature search was performed across PubMed, Scopus, and Web of Science databases up to August 2025. The search strategy utilized a combination of controlled vocabulary and free-text keywords, including, but not limited to, terms such as thyroid hormone, triiodothyronine, thyroxine, cardioprotective strategies, myocardial infarction, infarct size, and post-ischemic cardiac remodeling. Original experimental studies, clinical trials, meta-analyses, and high-quality mechanistic reviews published in English were evaluated for inclusion based on their relevance to the objectives of this review. Additional references were obtained through manual citation tracking of relevant articles.

## Cardioprotective therapies for STEMI with promising perspectives: current evidence

Therapy strategy recommendations to further reduce myocardial injury and subsequent cardiac remodeling in patients with STEMI have plateaued. However, there are several promising novel therapies that target different aspects of the ischemic process that have recently emerged, while older attempts have also shown positive but mostly conflicting results.

Early studies showed that Cyclosporine-A, an immunosuppressive agent that inhibits the mitochondrial permeability transition pore (mPTP) opening, could reduce infarct size. Subsequent larger trials failed to demonstrate improved clinical outcomes (11, 12). Glucagon-like peptide-1 (GLP-1) receptor agonists have emerged as promising agents in reducing infarct size due to their antioxidant and anti-inflammatory properties, which may mitigate endothelial dysfunction, but current studies have shown mixed results, not allowing implementation in clinical practice (13–16). In patients with STEMI and an increased risk of HF, treatment with Sodium-Glucose Co-Transporter 2 (SGLT2) inhibitors did not show to have a statistically significant impact on the composite outcome of cardiovascular death and first hospitalization for HF compared to the placebo (17, 18). However, in one study empagliflozin showed hopeful results as it was shown to contribute to a significantly higher left ventricular ejection fraction (LVEF) compared to placebo (19). Although nitrite serves as a source of nitric oxide (NO) and may protect the ischemic myocardium from reperfusion injury by reducing oxidative stress, antiplatelet activation, and inflammation, studies failed to show clinical benefits (20–22). Studies of colchicine, a well-known anti-inflammatory substance preventing subsequent inflammatory myocardial damage via NOD-like receptor protein 3 (NLRP3) inflammasome inhibition in patients with STEMI undergoing PCI, also published contradictory findings (23–26). Adenosine is a promising agent with cardioprotective capacities which involve an indirect anti-inflammatory effect and activation of protective intracellular signaling cascades (27, 28). Studies of prolonged adenosine intravenous delivery, as opposed to studies that used boluses or short infusions, resulted in a reduction of infarct size and better clinical outcomes in patients with anterior STEMI (29–31). Targeted anti-inflammatory treatment is also a promising field of research. Anakinra, a recombinant interleukin (IL)-1 receptor antagonist, has shown to have favorable effects on adverse left ventricular (LV) remodeling, reducing inflammatory markers and preventing new onset of HF after STEMI (32–34).

Interventions and procedures have yet to show consistent positive clinical outcomes. Remote ischemic conditioning (RIC), a non-invasive therapy that induces brief periods of ischemia and reperfusion in a remote tissue aiming to trigger systemic protective signaling pathways, has shown mixed results (35–38). Cooling of the myocardium is believed to have a cardioprotective effect after STEMI by lowering the metabolic demand and inhibiting inflammation. However, human trials have failed to confirm this benefit for all patients, with the exception of those with anterior STEMI who were successfully cooled to <35 °C (39–41). Pressure-Controlled Intermittent Coronary Sinus Occlusion (PICSO) uses a balloon-tipped catheter that periodically inflates and deflates in the coronary sinus, resulting in increased venous pressure and was found to improve coronary flow to ischemic zones (42). Findings from the recent, prematurely discontinued multicenter randomized PICSO AMI I trial indicated that PICSO, when used as an adjunct to PCI in patients with anterior STEMI, did not significantly reduce infarct size compared to conventional PCI alone and was associated with longer procedural time and greater contrast volume (43). Mechanical ischemic post-conditioning, a sequence of short ischemia/reperfusion cycles applied with an occluding balloon immediately after reperfusion of the infarct-related coronary artery, is thought to provide cardioprotection through the release of beneficial substances, activation of protective molecular pathways, and preservation of mitochondrial function (44, 45). While early human studies indicated a reduction in myocardial infarct size, subsequent research found no significant impact on all-cause mortality, re-infarction, or hospitalization for HF (46–50).

Finally, intracoronary supersaturated oxygen (SSO2) delivery was found to increase oxygen supply to viable myocardial tissue and improved microvascular flow, resulting in significant infarct size reduction (51). The AMIHOT II trial confirmed that infusion of SSO2 into the left anterior descending (LAD) artery among patients with anterior STEMI undergoing PCI within 6 hours of clinical presentation resulted in a significant reduction in infarct size (52). The subsequent IC-HOT trial further optimized SSO2 delivery technique, enhancing its safety and efficacy (53). This led to FDA approval of SSO2 therapy in anterior STEMI patients presenting within 6 hours of symptoms.

**Figure 1:**
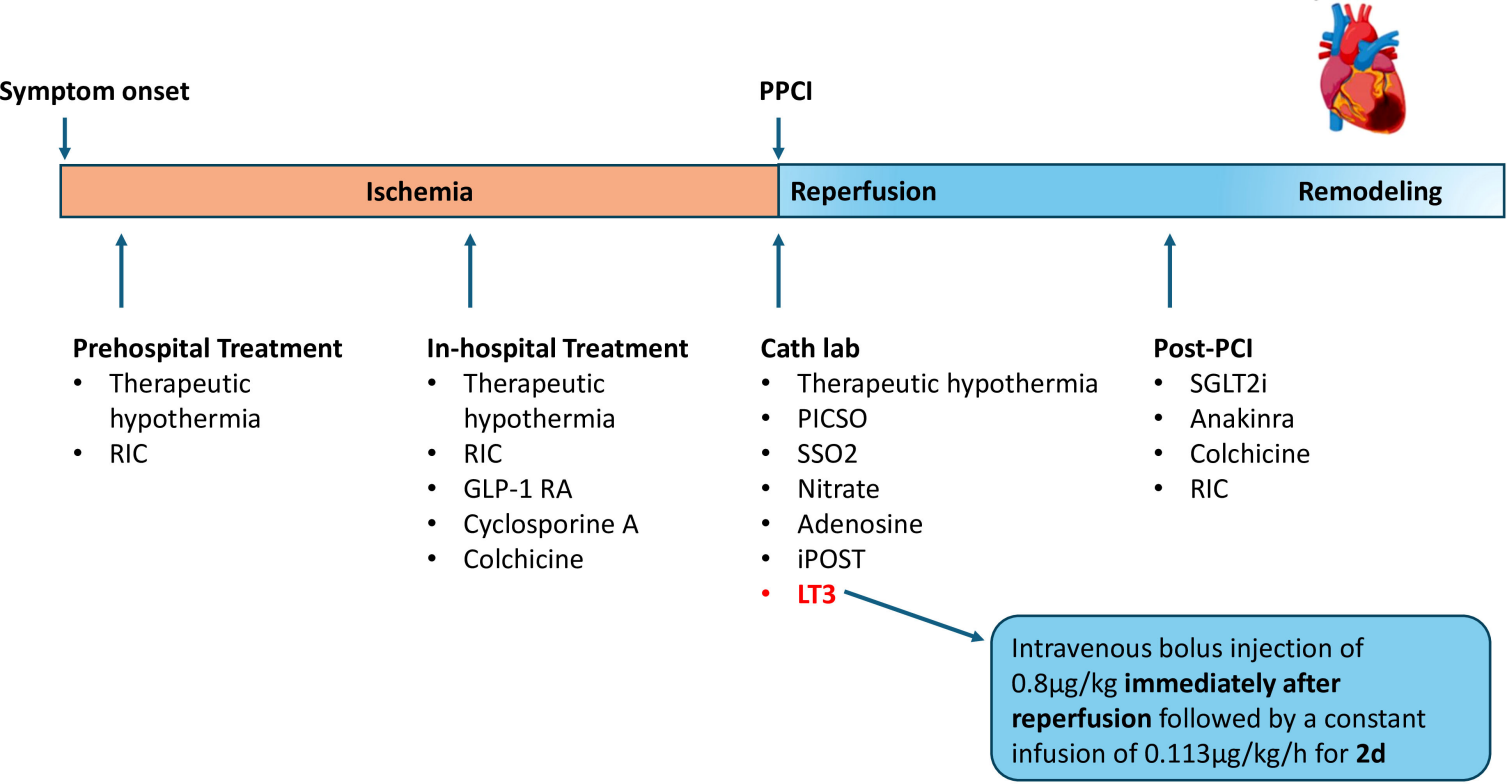
Diagram showing the different time windows during which cardioprotective interventions can be applied to limit myocardial infarct size in STEMI patients. STEMI, ST-elevation Myocardial Infarction; PPCI, Primary Percutaneous Coronary Intervention; RIC, Remote Ischemic Conditioning; GLP-1 RA, Glucagon-like peptide-1 Receptor Agonist; Cath lab, Catheterization Lab; PICSO, Pressure-Controlled Intermittent Coronary Sinus Occlusion; SSO2, Supersaturated Oxygen; LT3, Liothyronine; SGLT2i, Sodium-Glucose Transport Protein 2 Inhibitor; iPOST, Ischemic Postconditioning.

The above-mentioned interventions are summarized in **Table 1** and represent adjunctive therapies in the management of STEMI that have shown positive or mixed results in reducing infarct size and improving long-term outcomes. These therapies have often failed to achieve consistent clinical benefits due to several key challenges, including issues with the timing of administration, variability in patient factors such as age, comorbidities, and concomitant medications, as well as the complex mechanisms underlying reperfusion injury. Therefore, further large-scale clinical trials are essential to establish their potential role in routine clinical practice and in different sub-types of myocardial infarction (MI). **Figure 1** shows when these interventions are applied during the ischemia and reperfusion process.

**Table 1:** Myocardial protection strategies: mechanisms, outcomes, and clinical trials.

Drug/Device	Mechanism of action	Outcomes	Ongoing clinical trials to establish further effectiveness
Cyclosporine-A	Inhibits mitochondrial permeability transition pore, induces post-conditioning effect	Mixed results; A small study showed reduced infarct size (11). A larger study, failed to show a benefit (12).	N/A
GLP-1 Receptor Agonists	Antioxidant and anti-inflammatory effects	Mixed results with exenatide (14, 16). Liraglutide improved myocardial salvage and infarct size (15).	N/A
SGLT2 Inhibitors	Restores mitochondrial function, elevation of plasma levels of ketone bodies	No impact on cardiovascular death and first hospitalization for heart failure (17, 18). In one study, empagliflozin was associated with higher LV ejection fraction (19).	Short and Intermediate Term Effect of Dapagliflozin on Left Ventricular Remodeling in Anterior STEMI Patients (NCT05957887)
Nitrite	Source of nitric oxide, reduces oxidative stress	No overall benefit in reducing infarct size (20, 21); benefit in the subgroup of patients with thrombolysis in myocardial infarction flow ≤1 (22).	N/A
Colchicine	Inhibits NLRP3 inflammasome, reduces inflammation	Mixed results; one study showed benefit, two did not (23–26).	The Role of Colchicine in Reducing The Rate of Myocardial Reperfusion Injury (NCT05734612),
Adenosine	Activates protective intracellular signaling	Reduced infarct size and better outcomes in prolonged infusion studies (29–31).	N/A
Anakinra	IL-1 receptor antagonist, reduces inflammation	Positive effects on LV remodeling and heart failure prevention, reduced inflammation markers (32–34).	N/A
RIC	Triggers systemic protective signaling similar to post-conditioning	Mixed outcomes; two studies showed benefits (improved salvage index and long term outcomes, reduced cardiac remodeling) (36, 37), one larger had no impact on death and heart failure (38).	RIC-AFRICA (NCT04813159), RIP-HIGH (NCT04844931)
Therapeutichypothermia	Reduces metabolic demand and inflammation	No overall reduction in infarct size (41). Smaller infarcts in patients with anterior MI successfully cooled <35oC before PCI (40).	EURO-ICE (NCT03447834) (34)
PICSO	Improved coronary flow via increased venous pressure	PiCSO-AMI-I trial showed no benefit (43).	N/A
Mechanical Ischemic Post-Conditioning	Activates protective pathways post-reperfusion	Mixed outcomes (46–49); a *post-hoc* analysis suggested potential benefits in patients not receiving thrombectomy (50).	iPOST2 (NCT03787745)
Intracoronary SSO_2_	Increases oxygen supply and improves microvascular flow	Smaller infarcts in patients with anterior MI reperfused<6 h (52, 53).	N/A

GLP-1, Glucagon-like peptide-1; SGLT2, Sodium-Glucose Transport Protein 2; LV, Left Ventricle; STEMI, ST-elevation Myocardial Infarction; NLRP3, NOD-like Receptor Protein 3; IL-1, Interleukin-1; RIC, Remote Ischemic Conditioning; RIC-AFRICA, Remote Ischaemic Conditioning in STEMI Patients in Sub-Saharan Africa; RIP-HIGH, Remote Ischemic Conditioning With Local Ischemic Postconditioning in High-Risk ST-elevation Myocardial Infarction; MI, Myocardial Infarction; PCI, Percutaneous Coronary Intervention; EURO ICE, European Intracoronary Cooling Evaluation In Patients With ST-elevation Myocardial Infarction; PICSO, Pressure-Controlled Intermittent Coronary Sinus Occlusion; PiCSO-AMI-I, Pressure-controlled Intermittent Coronary Sinus Occlusion (PiCSO) in Acute Myocardial Infarction; iPOST2, Ischemic Postconditioning in STEMI Patients Treated With Primary PCI; SSO2, Supersaturated Oxygen.

## The prognostic role of thyroid hormone in acute ischemic myocardial damage: clinical observational studies

It has been long recognized that normal thyroid homeostasis often alters during an acute coronary event (54–56). Considering the direct association of thyroid function with the cardiovascular system, THs alterations are expected to have a significant prognostic role in patients with ACS. This notion is continuously confirmed by accumulating evidence which suggest a strong association between THs alterations in patients with ACS and adverse clinical outcomes. (**Table 2**).

**Table 2:** Summary of clinical studies on thyroid function and outcomes in acute coronary syndrome patients.

Study	Patients	Setting	Outcome
Lymvaios I et al., 2011 (64)	N=47	STEMI patients treated with PCI	T3 levels at 6 months predict cardiac functional recovery.
Lazzeri C etal. 2012 (62)	N=1047	STEMI patients treated with PCI	Lower fT3 levels in patients under 75 correlate with higher mortality.
Xue C etal. 2017 (65)	N=528	ACS patients treated with DES	Low fT3 levels predict worse HRQOL improvement.
Cao Q etal. 2018 (63)	N=1560	ACS patients treated with PCI	Low T3 syndrome is associated with 12-month adverse outcomes.
Gürdogan M etal. 2019 (66)	N=629	ACS euthyroid patients treated with PCI	High-normal TSH tertile predicts total mortality at 6 months.
Gao S etal. 2021 (67)	N=1162	Euthyroid patients with MINOCA	Lower fT3/fT4 ratio is associated with increased MACE risk.
Han C etal. 2022 (68)	N=1642	NSTE-ACS patients treated with PCI	Persistent SCH relates to severe coronary lesions and MACCE.
Li MF etal. 2022 (69)	N=1176	STEMI patients treated with PCI, CABG	SHyper and LT3S patients show higher in-hospital and long-term mortality than euthyroid patients.
Ni WC etal. 2023 (70)	N=1240	AMI patients with normal thyroid function	Third TSH tertile is linked to increased all-cause mortality risk.
Lang X etal. 2023 (71)	N=3648	AMI patients	FT3/FT4 ratio independently predicts heart failure and mortality.
Liu L etal. 2023 (72)	N=432	AMI patients with or without diabetes	Diabetic AMI patients with SCH have poorer in-hospital, 30-day, and long-term outcomes.

STEMI, ST-elevation Myocardial Infarction; T3, Triiodothyronine; FT3, Free Triiodothyronine; ACS, Acute Coronary Syndrome; DES, Drug-eluting Stent; HRQOL, Health-related Quality of Life; PCI, Percutaneous Coronary Intervention; TSH, Thyroid-stimulating Hormone; MINOCA, Myocardial Infarction with Non-obstructive Coronary Arteries; FT4, Free Thyroxine; MACE, Major Adverse Cardiac Event; NSTE-ACS, Non-ST elevation Acute Coronary Syndrome; SCH, Subclinical Hypothyroidism MACCE, Major Adverse Cardiac and Cerebrovascular Events; CABG, Coronary Artery Bypass Graft; SHyper, Subclinical Hyperthyroidism; LT3S, Low T3 Syndrome; AMI, Acute Myocardial Infarction.

Low-T3 syndrome stands out as one of the most prevalent thyroid findings among patients suffering from ACS (57, 58). This condition, also known as ‘non-thyroidal illness’ or ‘euthyroid sick syndrome’, refers to alterations of plasma concentrations in THs which occur during a variety of acute and chronic diseases in patients with no known intrinsic thyroid dysfunction (59–61). It is thought to be an adaptive and beneficial response which contributes in energy conservation following stress and is characterized by decreased triiodothyronine (T3) and/or free T3 (FT3), elevated reverse T3 (rT3) and normal thyroxine (T4) and thyroid-stimulating hormone (TSH) levels. This syndrome has been found to constitute an independent predictor of adverse outcomes and mortality in patients with ACS. In a series of 1, 047 patients with STEMI undergoing primary PCI, those with low-T3 syndrome had higher mortality compared to those with FT3 within the normal range (62). These observations were confirmed by another study that included 1, 560 ACS patients undergoing PCI. This study showed that low-T3 syndrome was associated with elevated overall 12-month adverse outcomes (63). Furthermore, T3 levels were found to be an independent factor of cardiac function recovery at 6 months post event and there was an association with impaired health-related quality of life (64, 65).

Even among patients with normal plasma concentrations of THs there is a strong association between thyroid dysfunction and adverse clinical outcomes. Thus, in a series of 1, 642 patients with NSTE-ACS undergoing PCI, persistent subclinical hypothyroidism (SCH) was associated with severe coronary artery lesions and major cardiovascular and cerebral events (MACCE), and its presence was proposed to be used as a predictor for evaluating the prognosis of these patients (68). Additionally, diabetic patients with acute MI (AMI) and SCH experienced worse in-hospital outcomes and higher risk of 30-day and long-term mortality (72). Patients with STEMI and subclinical hyperthyroidism were also found to have a higher incidence of adverse cardiovascular events and in-hospital mortality (69).

Even high–normal TSH levels (1.60-5.33 uIU/mL) are found to be associated with an increase in mortality during the 6-month follow-up period of euthyroid ACS patients (66). The association between TSH levels and mortality was also highlighted by a recent study in which euthyroid patients with AMI and TSH levels in the third tertile were at higher risk of all-cause mortality compared to patients who were in the first TSH-tertile subgroup (70). Single measurement of admission TSH levels in patients with coronary heart disease showed that patients with higher TSH levels had an increased risk of major adverse cardiac event (MACE) and all-cause mortality. Interestingly, the same study indicated that TSH levels in both the upper and lower reference range is associated with a risk of HF in these patients (73).

Furthermore, in a retrospective cohort study which included 8, 018 participants, free T4 (FT4) levels and FT3/FT4 ratio -often used to reflect the peripheral sensitivity of thyroid hormones- were found to be independent predictors of cardiovascular mortality and CVD disease (71). In the setting of AMI with nonobstructive coronary arteries, patients with lower FT3/FT4 ratio had worse prognosis with higher incidence of MACEs (67).

## Preclinical insights into the cardioprotective effects of thyroid hormone

About twenty years ago, Pantos et al. investigated the effects of TH pretreatment in an experimental model of ischemia-reperfusion (I/R) using isolated rat hearts. Their study showed that administration of TH confers protection against subsequent I/R injury, thereby improving the post-ischemic myocardial recovery in a pattern analogous to ischemic preconditioning (74). This cardioprotective effect involved inactivation of phospho-p38 mitogen-activated protein kinase (MAPK) and overexpression of protective molecules such as heat shock protein 27 (HSP27) and heat shock protein 70 (HSP70) (75–77). In similar I/R experimental models utilizing isolated rat hearts, T3 administration resulted in reduced apoptosis and subsequent improvements in cardiac function (78, 79). The investigators reported that the acute cardioprotective effect of T3 on I/R injury is mediated, at least partly, by the thyroid hormone receptors α1 (TRα1), which are overexpressed following an ischemic myocardial event, thereby enhancing regeneration, and by the suppression of the I/R-induced p38 MAPK activation. Of note, dose-response experiments with T3 treatment were not conducted and the heart rate was kept constant, which could have influenced the study’s outcomes (79).

Over the years, additional mechanisms of TH-mediated cardioprotection following acute ischemia have been highlighted. Fang et al. demonstrated that T3 administration in isolated rat hearts, at doses at least ten times the physiological limit, preserved calcium cycling proteins, such as sarcoplasmic/endoplasmic reticulum Ca2+ ATPase 2a (SERCA2a) and ryanodine receptor 2 (RyR2), and increased adenosine triphosphate (ATP) and creatine phosphate (CP) synthesis (80). In another isolated rat heart model of I/R investigating the post-conditioning effects of T3, when it was given at the onset of reperfusion resulted in improved post-ischemic myocardial function, with enhanced activation of PINK1-dependent mitophagy identified as a contributing mechanism (81). Post-conditioning may have greater clinical utility than pre-conditioning.

In one of the first studies of this kind, Pantos et al. explored whether TH administered shortly after an experimental model of AMI in rats could prevent adverse cardiac remodeling. In this study, rats subjected to coronary artery ligation were randomly divided, 24 hours post-operation, to either receiving food containing T3 and T4 for two weeks (AMI-THYR group) or not (AMI group), while sham-operated rats served as controls. TH administration was associated with a significantly higher LVEF% (45.8% for the AMI-THYR group vs. 30.0% for the AMI group, p<0.05) and significantly smaller LV internal diameters at both diastolic (LVIDd) and systolic (LVIDs) phases compared to the AMI group (LVIDd 8.8mm for the AMI-THYR group vs. 9.2mm for the AMI group, p=0.035, LVIDs 7.1mm for the AMI-THYR group vs. 8.1mm for the AMI group, p=0.001) (82). A potential limitation of this study is that the observed improvement in functional parameters may partly reflect the peripheral vasodilatory action of TH, which can reduce afterload. Therefore, load-dependent measures such as LVEF might overestimate the true myocardial effect of TH. Improved LVEF and ellipsoidal reshaping of the LV were also reported following longer-term TH administration (13 weeks) in a comparable experimental rat model of AMI (83).

Numerous other animal models of infarct-related myocardial injury have consistently demonstrated the cardioprotective role of TH (84–89). These studies indicated that T3 administration following AMI promotes favorable changes in various pathophysiological pathways, including the expression of myosin heavy chains (decreased β-MHC and increased α-MHC), T3-dependent miRNA-gene interactions, calcium cycling proteins, pro-survival signaling (such as Akt) and the prevention of mitochondrial impairment (84–89).

TH also exerts antioxidant, antifibrotic, and pro-angiogenic mechanisms. Oxidative stress is characterized by the accumulation of reactive oxygen species (ROS) and is considered a major contributor to IRI following myocardial reperfusion (90, 91). Experimental data showed that TH lowers ROS and lipid peroxidation, suppresses nicotinamide adenine dinucleotide phosphate (NADPH) oxidase activity, and normalizes the glutathione (GSH) to oxidized glutathione (GSSG) ratio, with even stronger effects when combined with beta-blockers (92, 93). Moreover, TH inhibits fibrosis by modulating antifibrotic micro ribonucleic acid (miRNA) signaling disrupted by prolonged transforming growth factor-β1 (TGFβ1) activity, and enhances angiogenesis via integrin αVβ3-mediated activation of extracellular regulated kinase (ERK) signaling, and by activating the phosphatidylinositol 3’ -kinase (PI3K) pathway through cytoplasmic thyroid hormone receptor beta (TRβ), which induces hypoxia-inducible factor-1α (HIF-1α) expression and vascular growth (94, 95).

Moreover, mitochondrial dysfunction plays a key role to the pathogenesis of IRI and post-ischemic cardiac remodeling. TH supports mitochondrial function by inhibiting p53 signaling, reducing mitochondrial oxidative stress, preventing mPTP opening, enhancing mitochondrial biogenesis, and regulating cardioprotective miRNAs (96). A relatively recent study has attributed TH’s mitochondrial favorable effects after IRI to the activation of the reperfusion injury salvage kinase (RISK) pathway, when it is given early and at high doses (97). Another study using a rat model of cryoinjury-induced MI showed that acute T3 administration following myocardial injury not only limits scar expansion but also preserves mitochondrial integrity over the long term by inhibiting the accumulation of mitochondrial-damaging long-chain acylcarnitines. These findings are strengthened by the use of the cryoinjury model, which produces a highly reproducible, transmural LV necrosis with a uniform scar, superior to the variable infarcts seen with left anterior descending artery (LAD) ligation. This consistency allows for more reliable evaluation of myocardial repair, making the study’s conclusions more robust (98).

**Figure 2:**
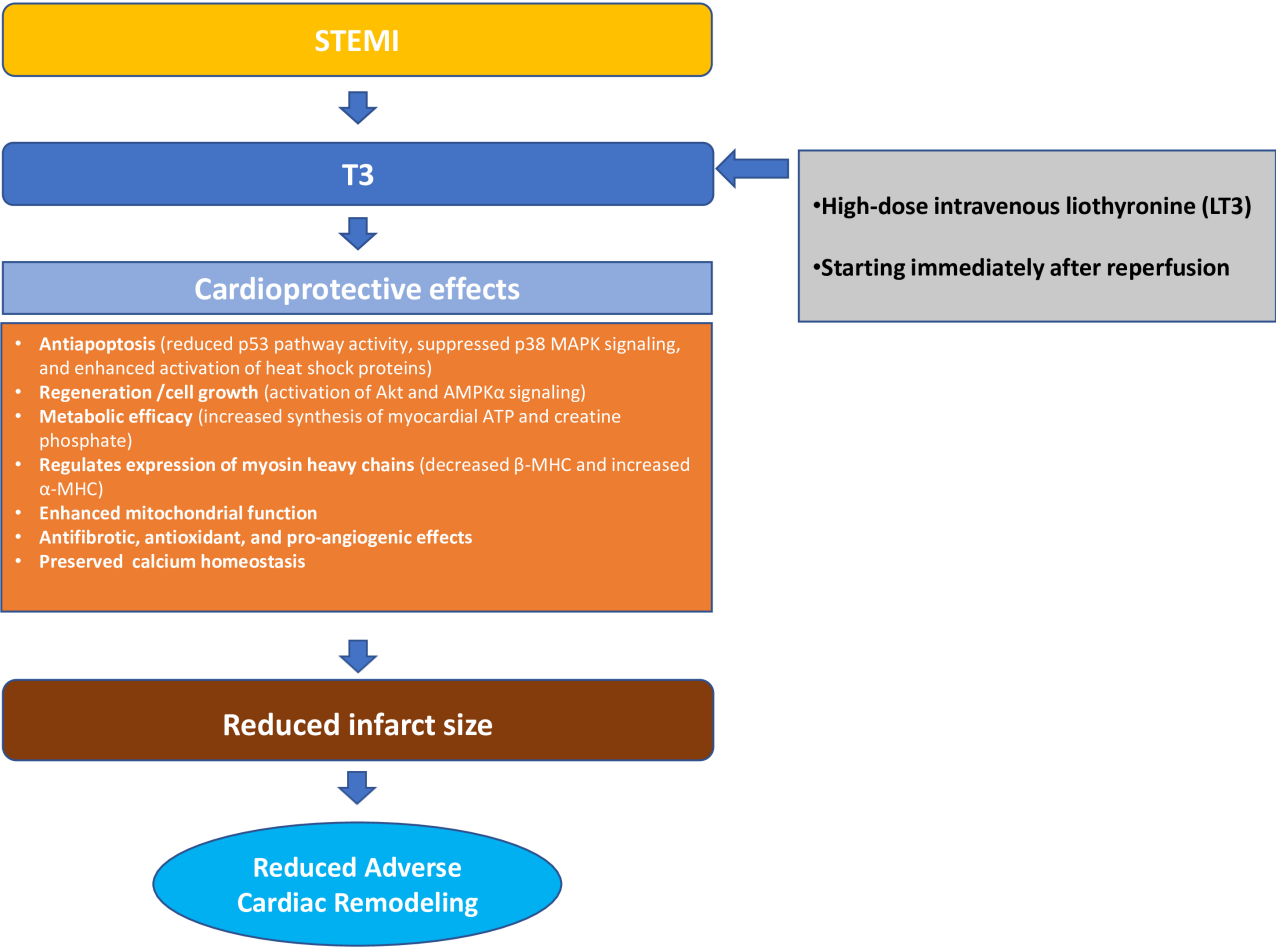
Cardioprotective effects of T3 after STEMI. STEMI, ST-elevation Myocardial Infarction; T3, Triiodothyronine; p38 MAPK, Phospho-p38 Mitogen-activated Protein Kinase; AMPKα, AMP-activated Protein Kinase α; ATP, Adenosine triphosphate; β-MHC, β-myosin heavy chain; α-MHC, α-myosin heavy chain.

The effects of TH on cardiac remodeling after MI are dose- and time-dependent and remain effective in the presence of co-morbidities such as diabetes (79, 82, 83, 99, 100). Notably, diabetes almost doubles mortality after MI (101). In diabetic animals, TH administration has been shown to improve cardiac function via the increased activation of both Akt and AMP-activated protein kinase α (AMPKα) signaling (100, 102). (**Figure 2**).

## Clinical perspectives on the cardioprotective role of thyroid hormone in STEMI

The aforementioned preclinical trials have paved the way for clinical trials investigating TH administration in STEMI patients (**Table 3**). A phase II study conducted by Pingitore et al. examined the impact of TH replacement therapy on cardiac function and infarct size in patients with AMI and low-T3 syndrome. This study enrolled 37 patients with STEMI who were randomized to receive T3 for six months or not. Primary endpoints (LVEF, LV volumes, wall motion score index, infarct size) were measured using cardiac magnetic resonance (CMR) imaging at discharge and at six months. Although TH replacement therapy improved regional contractile dysfunction and stroke volume at six months, it had no effect in infarct size, LV volumes and LVEF. Of note, the investigators selected low doses of T3 to minimize the risk of adverse effects. While this strategy likely contributed to the favorable safety profile of the regimen, it may also have resulted in T3 levels insufficient to elicit its potential cardioprotective actions (103).

**Table 3:** Clinical studies with TH administration in patients with AMI.

Study/first author	Publication year	Design	Treatment duration	Total follow-up	Participants	Treatment	Comparator	Outcomes
Pingitore et al. (103)	2019	Phase II, randomized controlled clinical trial	6 months	6 months	37 STEMI patients with LT3S	T3 replacement therapy (maximum dosage 15 mcg/m2/die).	Placebo	Significant reduction in WMSI difference value (discharge/follow-up), higher stroke volume at follow-up.
Jabbar et al. (104)	2020	Double-blind, randomized clinical trial	52 weeks	52 weeks	95 STEMI/NSTEMI patients with subclinical hypothyroidism	Daily levothyroxine (25 μg titrated to aim TSH levels between 0.4 -2.5 mU/L).	Placebo	Levothyroxine treatment safe; no significant improvement in LVEF, LV volumes, or infarct size.
ThyRepair Study (105)	2022	Pilot, randomized, double-blind, placebo-controlled trial	48 hours	6 months	37 acute anterior STEMI patients	i.v. bolus injection of 0.8 μg/kg of LT3 followed by a constant infusion of 0.113 μg/kg/h i.v. starting immediately after reperfusion.	Placebo	Significantly lowerLVEDVi and LVESVi at discharge, IV tended to be lower in the LT3-treated group at 6 months, no significant differences in LVEF%
Pantos et al. (106)	2024	*Post-hoc* analysis of data from the ThyRepair study	48 hours	Until discharge (aprox. 7 days)	41 acute anterior STEMI patients	i.v. bolus injection of 0.8 μg/kg of LT3 followed by a constant infusion of 0.113 μg/kg/h i.v. starting immediately after reperfusion.	Placebo	LT3 treated patients with IV > 20% had significantly higher LVEF% and lower and LVESVi at discharge. No significant differences forpatients with IV ≤ 20%.

AMI, Acute Myocardial Infarction; STEMI, ST-elevation Myocardial Infarction; LT3S, Low T3 Syndrome; T3, Triiodothyronine; WMSI, Wall Motion Score Index; LV, Left Ventricle; LVEF, Left Ventricle Ejection Fraction; NSTEMI, Non ST-elevation Myocardial Infarction; TSH, Thyroid-stimulating Hormone; LVEDVi, Left Ventricle End-diastolic Volume Index; LVESVi, Left Ventricle End-systolic Volume Index; LT3, Liothyronine; IV, Infarct Volume.

The potential effects of TH therapy on cardiac function following AMI were further investigated in a double-blind placebo-controlled study that included 95 STEMI and non-STEMI (NSTEMI) patients who had subclinical hypothyroidism. The patients were randomized to receive either a daily capsule of levothyroxine (starting dose at 25 μg titrated to serum TSH levels between 0.4 and 2.5 mU/L) or an identical placebo for 52 weeks. The primary outcome measure was LVEF, assessed by CMR at baseline and at the end of the study. The investigators concluded that while levothyroxine treatment was safe, it did not significantly improve LVEF after 52 weeks compared to placebo, nor did it impact LV volumes or infarct size (104).

Several factors may explain these negative results. During ischemic stress, an intra-cardiomyocyte hypothyroid state occurs due to changes in deiodinase activity which impairs the conversion of T4 to T3. Further, alterations to the level of TH receptors take place which modify the myocardium’s response to THs, suggesting that higher doses of T3 might be necessary to restore intra-myocardial TH levels (107). Furthermore, the study’s heterogeneous patient population included nearly 40% of individuals with preserved LVEF, which could mask treatment efficacy, as minimal myocardial infarction size typically does not lead to significant cardiac remodeling. Lastly, since the cardiac remodeling process starts early after reperfusion, the timing of TH administration is likely to have a crucial role in its healing response (107). Probably, the delay between the diagnosis of AMI and initiation of T4 (median 17 days) had exceeded the therapeutic window.

The ThyRepair study was a pilot, double-blind, placebo-controlled trial of 37 acute anterior STEMI patients who were randomized immediately after successful reperfusion to receive either high-dose intravenous liothyronine (LT3) treatment or placebo for 48 hours (105). Specifically, a 3′, 5-triiodo- L-thyronine sodium solution for injection with concentration 10μg/ml (T3^®^ Solution, Uni-Pharma Pharmaceutical Laboratories S.A., Kifissia, Greece) was administered as an intravenous bolus injection (0.8 μg/kg) followed by a constant infusion of 0.113 mg•kg−1•h−1 I.V. for 48 hours using a pump. The placebo group received equivalent volumes of the vehicle with an identical composition, apart from the active substance. In the LT3-treated group, total T3 levels increased approximately sevenfold for 48 h compared to the placebo group. This study recruited subjects to up to 12 hours total ischemic time. Cardiac function, remodeling, and infarct volume were assessed using CMRI at hospital discharge and six months follow-up. The primary endpoint was LVEF at six months post-infarction, while the main prespecified secondary outcomes were LV volumes, infarct volume and safety at discharge and 6 months follow-up (105).

Regarding the primary endpoint, acute treatment with LT3 did not significantly improve LVEF compared to placebo at hospital discharge and 6-month post-infarction. However, CMR LVEF difference between groups was at the magnitude of 5 U in favor of LT3-treated group (49.1 ± 8.4% in the LT3-treated group and 44.2 ± 10.2 in the placebo group at discharge, p=0.11, 53.6 ± 9.5% in the LT3-treated group and 48.6 ± 11 in the placebo group at 6 months, p=0.15). This improvement is significant, as an increase in LVEF greater than 5 units can have beneficial effects on mortality and the incidence of new-onset HF (108). It appears that two factors contributed to not achieving statistically significant difference in LVEF%, the small sample size and that LVs with higher dilatation, which untreated patients had, benefit from Starling’s law effect and its positive effect in contractility (105).

In this study, CMR LV end-diastolic volume and CMR LV systolic volume index were significantly lower in the LT3-treated group compared to the placebo group at hospital discharge [left ventricle end-diastolic volume index (LVEDVi) 92.2–16.8mL/m2 vs. 107.5–22.2, p=0.022 and left ventricle end-systolic volume index (LVESVi) 47.5–13.9mL/m2 vs. 61.3–21.7, p=0.024, respectively], indicating a potential positive effect of acute LT3 treatment on early LV chamber remodeling (LV dilatation). These findings may be of clinical importance, as early LV dilatation is correlated with worse clinical outcomes compared to late and no LV dilatation (109). Further, CMR infarct volume tended to be lower in the LT3-treated group (18.7 ± 9, 5 ml vs. 25.9 ± 11.7 in placebo group, p=0.05) at 6 months, exposing a possible late cardioprotection effect of T3. An additional interesting finding was that the ECG QRS duration was significantly lower in the LT3-treated group as compared with placebo at 6 months follow-up (87.2 ± 7msec vs. 96.3 ± 15, p<0.05, respectively). It is well established that prolonged duration of QRS complex after AMI is associated with increased mortality (110).

The investigators of the ThyRepair study concluded that the administration of high-dose T3 in a post-acute MI setting was safe and well-tolerated. However, a modest increase in heart rate and a tendency for a higher incidence of atrial fibrillation were observed in LT3-treated patients during the first 72 hours of hospitalization. Neither of these findings resulted in adverse effects on cardiac function or injury in the study population, but they could pose potential risks in the general population, particularly in those with more comorbidities and extensive coronary artery disease. Important to note, serious ventricular arrhythmias (ventricular fibrillation or sustained ventricular tachycardia) were not observed in either group during hospitalization. In addition, 30% of patients in the LT3 group experienced transient episodes of elevated temperature (>37.8 °C), likely due to the hypermetabolic effects of T3. These episodes were mild and resolved with paracetamol. LT3-treated patients occasionally reported mild nervousness, which was transient and did not require intervention.

Based on the findings of the ThyRepair study, T3^®^ Solution for injection was approved by the FDA for use in larger studies to further explore its effect on cardiac remodeling following STEMI.

More recently, a *post-hoc* analysis of data from the ThyRepair study was conducted to explore whether the effects of acute LT3 treatment on post-infarcted myocardium depend on the severity of infarct size (106). In this analysis, 41 anterior STEMI patients from the ThyRepair study were divided into two groups based on the median value of CMR infarct volume (IV): one group with small infarct size (CMR IV<20%, group A) and another with large infarct size (CMR IV>20%, group B). Interestingly, in group B, acute high-dose LT3 treatment resulted in significant improvement in CMR LVEF% (47.3 ± 6.5 mL/m² vs. 39.9 ± 8.7 mL/m² for placebo, p < 0.05) and smaller LV volumes (LVEDVi and LVESVi were 91.8 ± 18.6 mL/m² and 49.0 ± 14.0 mL/m² vs. 112 ± 23.8 mL/m² and 68.3 ± 21.5 mL/m² for the placebo group, p <0.05) at discharge. Conversely, in group A, the placebo and LT3-treated groups had similar LVEF% (56.8 ± 10.2 mL/m² vs. 52.2 ± 10.5 mL/m²), LVEDVi (90.9 ± 19.8 mL/m² vs. 92.8 ± 14.5 mL/m²), and LVESVi (40.8 ± 18.2 mL/m² vs. 44.9 ± 14.1 mL/m²) at discharge. Furthermore, in group B, CMR left ventricular mass index (LVMi) was lower in T3-treated patients vs. placebo but did not reach statistical significance (p = 0.1). Microvascular obstruction (MVO) was 1.9 ± 2.2 in placebo vs. 0.84 ± 0.9 in the LT3-treated group (p = 0.15).

The present *post-hoc* analysis of the ThyRepair study suggests that acute high dose LT3 treatment may exert more favorable effects on the recovery of cardiac function in patients with large infarct sizes and worse prognoses. Furthermore, it highlights a potential effect of LT3 on myocardial edema and microvascular obstruction. These novel findings merit further investigation in larger clinical trials.

## Discussion

Cardiac remodeling is a process that begins early after ischemia and is distinguished by the reactivation of the fetal transcriptional program (111). Heart repair after AMI engages the same regulatory networks that are involved in embryonic cardiac development. TH, an evolutionarily conserved hormone, is a key modulator of these developmental pathways and is positioned as a biologically aligned candidate that can promote myocardial repair (105, 111).

As outlined in this review, numerous animal studies have shown that TH can promote post-ischemic myocardial repair by modulating differentiation programs, activating pro-survival pathways, enhancing mitochondrial function and metabolic processes, inhibiting apoptosis, regulating inflammatory activation, and reducing fibrosis. These mechanisms exert favorable effects on infarct size and cardiac remodeling. However, despite these promising preclinical data, translating TH to humans poses challenges due to species-specific physiological differences. TRα1 is present in both rodents and humans, but thyroid hormone receptor α2 (TRα2) is absent in rat and mouse hearts while present in human hearts, and thyroid hormone receptor β1 (TRβ1) is detected in rodents but not in human hearts (112). In addition, differences in myosin heavy chain (MHC) isoform expression and cardiac deiodinase activity may further alter the cardiac response to T3, highlighting the need for caution when extrapolating rodent data to humans (113, 114). Nevertheless, the ability of TH to act on several key pathophysiological mechanisms underlying infarct progression, unlike other proposed cardioprotective interventions, confers a unique therapeutic profile.

Translating TH into clinical practice has also been approached with considerable skepticism, reflecting the long-standing belief that TH could be harmful in the setting of ischemia by increasing heart rate and myocardial oxygen demand. Moreover, there are concerns regarding the endocrine consequences of T3 administration in patients with an intact thyroid axis. However, in the modern era of reperfusion therapy, its role in I/R injury has been reconsidered. Indeed, the beneficial effects of T3 on post-ischemic cardiac performance are not associated with increased myocardial oxygen consumption or heightened myocardial injury, as demonstrated in both animal models and in patients undergoing bypass surgery, AMI, or treatment for chronic HF (105, 115, 116). Furthermore, based on the findings of the ThyRepair study, TSH levels were significantly lower in the LT3 group, while TSH levels were similar between the two groups at admission and discharge. Additionally, there were no statistically significant differences in total T3, total T4, or TSH between the two groups at 3 and 6 months, indicating that LT3 administration did not cause any long-term thyroid dysfunction (105). However, given the small number of patients, these findings should be interpreted with caution.

Thus far, two small clinical trials have confirmed the safety of chronic T3 and T4 administration in patients who suffered AMI but failed to reach their primary endpoints (103, 104). The ThyRepair study also failed to meet its primary endpoint of significantly improving LVEF, likely due to the limited sample size. However, it demonstrated signals of potential benefit, including attenuation of acute cardiac dilation and improved IV at 6 months. It also defined the optimal process for TH administration to achieve maximal cardioprotective benefits. High doses of intravenous LT3 delivered immediately after reperfusion, followed by a 2-day constant infusion, appear to be most effective (105). Proceeding further, a *post-hoc* analysis of the ThyRepair study showed that acute LT3 administration in anterior STEMI patients with large infarct areas produced long-term improvements in LVEF and LV volumes, highlighting the potential benefit of LT3 in large infarcts where intensified treatment is most required (106).

LT3 administration also offers several practical advantages. It does not delay PPCI, requires no specialized equipment, follows a simple dosing regimen, is administered over a short period, and remains a low-cost intervention. All of these features make it a feasible and widely applicable therapeutic option. In addition, targeted nanoparticle-based delivery of T3 may offer a promising strategy to minimize systemic adverse effects while maintaining its cardioprotective benefits. Lastly, LT3 can be combined with other cardioprotective strategies. For instance, techniques that enhance coronary flow (e.g., PICSO) may deliver LT3 more effectively to ischemic cardiomyocytes and potentially enhance its therapeutic efficacy.

## Conclusion

The search for effective cardioprotective therapies for STEMI remains in central focus in cardiovascular medicine. As discussed, numerous strategies are being actively explored, including novel pharmacological agents and mechanical interventions, which could offer additional protection to the myocardium during ischemia and reperfusion. The combination of these diverse therapies represents an exciting area of future research and may offer a more comprehensive solution to improved outcomes for STEMI patients.

In the case of TH, the preclinical and clinical studies completed to date have demonstrated that TH, when given in the right time and in the right dosage, is a promising cardioprotective agent to use with timely reperfusion to reduce early LV chamber remodeling, improve LVEF, and limit infarct size in patients with STEMI, especially those with large infarct size. However, the cardioprotective effects of TH observed in preclinical models of AMI have yet to be consistently demonstrated in clinical trials. The small number of patients in these clinical studies must be considered, and the data need validation through larger RCTs.
